# Trend of Urban-Rural Disparities in Hospital Admissions and Medical Expenditure in China from 2003 to 2011

**DOI:** 10.1371/journal.pone.0108571

**Published:** 2014-09-24

**Authors:** Rong Fu, Yupeng Wang, Han Bao, Zhiqiang Wang, Yongquan Li, Shaofei Su, Meina Liu

**Affiliations:** 1 Department of Biostatistics, Public Health College, Harbin Medical University, Harbin, PR China; 2 School of Medicine, the University of Queensland, Royal Brisbane & Women’s Hospital, Herston, Australia; 3 Graduate school, Harbin Medical University, Harbin, PR China; UNAIDS, Trinidad and Tobago

## Abstract

**Objectives:**

To assess the trend of urban-rural disparities in hospital admissions and medical expenditure between 2003 and 2011 in the context of Chinese health-care system reform.

**Methods:**

The data were from three different national surveys: the Third National Health Services Survey in 2003, the Fourth National Health Services Survey in 2008 and the national health-care reform phased assessment survey in 2011. There were 151421, 143380 and 48356 respondents aged 15 years or older in 2003, 2008 and 2011, respectively.

**Results:**

The health insurance coverage expanded considerably from 27.7% in 2003 to 96.4% in 2011 among respondents aged 15 years or older. Hospitalization rate increased rapidly from 4.1% in 2003 to 9.6% in 2011. Urban respondents had higher hospital admissions than rural respondents, and the *RR* (95% *CI*) of hospitalization was 1.23 (1.17–1.30), 1.06 (1.02–1.10) and 1.16 (1.10–1.23) in 2003, 2008 and 2011, respectively. The urban-rural disparity in hospital admissions significantly narrowed over time. Urban respondents had a higher admission rate if insured and a lower admission if not insured than their rural counterparts. Of the six medical expenditure measures, the disparities in reimbursement rate and the proportion of hospitalization direct cost to the total consumer spending significantly narrowed.

**Conclusions:**

The health insurance coverage has been continually expanding and health service utilization has been substantially improved. Urban-rural disparities have been narrowed but still exist. Therefore, policy-makers should focus on increasing investment and reimbursement levels, developing a uniform standard health insurance system for urban and rural residents and improving the medical assistance system.

## Introduction

The epidemiological transition shifted the predominant causes of mortality from infectious to chronic diseases over the last few decades in China. The rapidly increased morbidity and mortality of chronic diseases have posed a huge burden of disease to patients and society. [1], [2], [3], [4] In 2001, the total health expenditure in China was 502.5 billion Renminbi (RMB, $1 = 6.11RMB) and 60% of it was paid by patients themselves. [5], [6] Most medical resources were allocated to urban areas while poor rural residents could not afford expensive medical expenditure resulting in the inequity of health service utilization among urban and rural residents. [7] It was reported that health service was underused by respondents who reported illness in the two weeks before the survey. [8] There was a large disparity in hospital admissions between urban and rural respondents with 11.1% hospitalization rate in urban area versus 7.6% in rural area. The per capita hospital costs associated with hypertension, diabetes, coronary heart disease, stroke and cancer were around 4000–10000 RMB, in comparison with the average annual income of 9061.2 RMB for an urban family and 3582.4 RMB for a rural family in 2003. [8], [9], [10] It was thus clear that the burden of disease was much higher for rural than for urban residents.

The Chinese health-care system had been strongly impacted by Severe Acute Respiratory Syndromes (SARS) in 2003. [11] The government looked back at the current health-care system and realized that it was in great need of increasing input to public health and medical institutions to fundamentally reform the health-care system. In addition to Urban Employee Basic Medical Insurance (UEBMI) which was developed in 1998, [12], [13] New Rural Cooperative Medical Scheme (NRCMS) and Urban Resident Basic Medical Insurance (URBMI) were promulgated by the State Council in 2003 and 2007 respectively. [14], [15], [16], [17] Both URBMI and NRCMS aimed to expand insurance coverage in the initial stages of reform. The government increased investments up to 1.51 trillion RMB between 2009 and 2011 to deepen the health-care system reform. [18].

In the context of recent health-care system reform, the trend of urban-rural disparities in health service utilization was an attentive hotspot for health service researchers and policy makers. Previous studies about urban-rural disparities in health service utilization were limited to a specific area or a province in China and using the statistical methods which neglected the hierarchical nature of the data of National Health Service Survey (NHSS). [19], [20], [21], [22], [23], [24] Furthermore, there was little empirical evidence on the trend of urban-rural disparities in health service utilization. To fill this knowledge gap, we assessed the 8-year trend of urban-rural disparities in hospital admissions and medical expenditure at the national level using the data from the China Third NHSS in 2003, the Fourth NHSS in 2008 and the national health-care reform phased assessment survey in 2011. In this study, we analyzed the gains achieved by the Chinese health-care system reform and identified the existing problems from health service perspectives to provide evidence for improving the accessibility and quality of health service for Chinese residents.

## Method

### Data source

The multiple data sets were used including the China Third NHSS in 2003, the Fourth NHSS in 2008 and the national health-care reform phased assessment survey in 2011. The NHSS, the largest statewide health survey in China, had been conducted by Ministry of Health every 5-year since 1993 and collected data on multiple public health issues including health status, health behaviors, access to healthcare, and healthcare utilization. All of these surveys were done with a multistage cluster sampling method to randomly select the sample. Among 2859 counties in 31 provinces or autonomous regions except Hong Kong, Macau and Taiwan, 94 counties were randomly selected as sample areas (95 counties in 2003). In each county, 5 townships were randomly selected and two villages or neighborhood committees were randomly selected in each township. In 2003 and 2008, 60 households from each village or neighborhood committee were randomly selected and 20 households in 2011. The eligible respondents were all family members in each household.

Study participants were interviewed by local medical workers with a structured questionnaire supervised by the doctors from township hospitals or higher-level health institutions. During the survey, if a selected household was not interviewed up to three attempts on different days, it would be replaced by a candidate household. The interviewers first explained the purposes of the survey and ensured the confidentiality of study participants. All participants who gave oral informed consent to participate in the survey were interviewed. The Ethics Committee of China’s Ministry of Health approved this consent procedure. To insure the quality of data, a district survey manager checked the completeness of questionnaires at the end of each day. If there were missing information or logic errors, individuals were re-interviewed at the next day. After the survey, a 5% sample of the households was randomly selected and resurveyed with eight questions to examine survey quality. The consistency between survey and resurvey was 95%.

### Study population and Measures

A total of 193689 respondents from 57023 households in 2003 (177501 from 56456 in 2008, and 59835 from 18822 in 2011) were interviewed. The response rate was 98.3%, 95.0% and 95.5% in 2003, 2008 and 2011, respectively. We excluded respondents who were aged <15 years old and those with missing values. This resulted in a total of 151421 respondents in 2003, 143380 in 2008 and 48356 in 2011. Demographic variables included sex, age, marital status, education, residential area, geographic region, health insurance status (insured or not), and distance between home and the closest medical center.

Hospitalization within the last one year before the survey and hospitalization costs were chosen to measure the changes of urban-rural disparities in hospital admissions and medical expenditure. Hospital admissions were confirmed based on the question, “How many times did you hospitalize during the last one year?” If a respondent hospitalized, the reason would be recorded, such as illness, delivery, injury/poisoning, recovery, birth control, et al. Hospitalization rate was calculated as the total number of admissions divided by the total number of respondents.

Hospitalization costs were determined based on the following three questions, (1) “How much did you pay for this hospitalization?” (2) “How much reimbursement did you get for this hospitalization?” (3) “How much did you spend other than hospitalization cost, such as travelling, board and care expense?” The first one was defined as hospitalization direct cost and the last as indirect cost. Reimbursement rate was calculated as the reimbursement amount divided by the hospitalization direct cost. To make the comparison of medical expenditure across time meaningful, the amount of all medical expenditure measures in 2008 and 2011 were transformed by Consumer Price Index (CPI) to the price level in 2003. The transformation formula is following: real price = nominal price × (CPI of base year/CPI of object year). The CPI was 438.7, 522.7 and 565.0 in 2003, 2008 and 2011, respectively, reference as CPI = 100 in 1978. [6] Therefore, the real price of 100 RMB which was nominal price was transformed to 83.9 RMB [100 RMB × (438.7/522.7)] in 2008 and 77.6 RMB [100 RMB × (438.7/565.0)] in 2011.

### Statistical analysis

Numbers (percentages) were reported for categorical variables. *P* values for differences between three surveys were not reported because of the large sample size. Means were calculated for medical expenditure measures and the trend over time was examined with Analysis of Variance (ANOVA). The difference and 95% confidence interval in medical expenditure between urban and rural respondents were calculated and tested using Student’s *t* test. Because individuals clustered within each household, General Estimating Equations (GEE) with log link were used to assess the trend of hospital admissions from 2003 to 2011 and to estimate the rate ratio (*RR*) and 95% confidence interval (*CI*) of hospitalization for urban versus rural respondents. [25], [26] Stratified analysis by health insurance status was conducted to determine whether the changes of disparities differed across strata. All analyses were conducted using SAS version 9.1. A two-sided *P*<0.05 was established as the level of statistical significance for all tests.

The data was stored on password-protected computers at the centre for health statistics information of Ministry of Health in Beijing. To protect the confidentiality of study participants, only de-identified data were available to the researchers of this project. The data was analyzed anonymously.

## Results

### Demographic Characteristics

Demographic characteristics for the respondents are shown in Table 1. The sex ratio was balanced. The percentage of respondents who were aged older than 55 years increased rapidly, from 23.6% in 2003 to 34.1% in 2011. More and more people had higher education, but the proportion of people who had a university degree or higher was still relatively low. Rural respondents accounted for most of the population, approximately 72%. In total, 96.4% of respondents had health insurance in 2011 but only 27.7% in 2003. The types of health insurance included UEBMI, URBMI, NRCMS, free medical service, commercial medical insurance, et al. About 90% of respondents lived within 3 kilometers to the closest medical center.

**Table 1:** Demographic characteristics of the respondents from three national surveys.

Variables	2003	2008	2011
	N(% of 151421)	N(% of 143380)	N(% of 48356)
Sex			
Male	75476 (49.8)	70529 (49.2)	23820 (49.3)
Female	75945 (50.2)	72851 (50.8)	24536 (50.7)
Age (year)			
15–24	25479 (16.8)	20672 (14.4)	6308 (13.0)
25–34	27520 (18.2)	19830 (13.8)	6684 (13.8)
35–44	32365 (21.4)	30685 (21.4)	9155 (18.9)
45–54	30280 (20.0)	28762 (20.1)	9718 (20.1)
55–64	17196 (11.4)	22428 (15.6)	8886 (18.4)
≥65	18581 (12.3)	21003 (14.6)	7605 (15.7)
Marital status			
Unmarried	27164 (17.9)	23398 (16.3)	7544 (15.6)
Married	113339 (74.9)	107790 (75.2)	36926 (76.4)
Divorced	1594 (1.1)	1953 (1.4)	648 (1.3)
Widow	9324 (6.2)	10239 (7.1)	3238 (6.7)
Education			
Elementary school or lower	68715 (45.4)	60146 (41.9)	18971 (39.2)
Junior high school	51859 (34.2)	51057 (35.6)	17883 (37.0)
Senior high school	22330 (14.7)	22866 (15.9)	7721 (16.0)
Junior college	4891 (3.2)	5073 (3.5)	1933 (4.0)
University or higher	3626 (2.4)	4238 (3.0)	1848 (3.8)
Residence area			
Urban	41441 (27.4)	39811 (27.8)	13622 (28.2)
Rural	109980 (72.6)	103569 (72.2)	34734 (71.8)
Geographic region			
Eastern China	51588 (34.1)	49750 (34.7)	16565 (34.3)
Mid-China	42393 (28.0)	40327 (28.1)	13665 (28.3)
Western China	57440 (37.9)	53303 (37.2)	18126 (37.5)
Health insurance status			
No	109416 (72.2)	15859 (11.1)	1742 (3.6)
Yes	42005 (27.7)	127521 (88.9)	46614 (96.4)
Distance between home and the closest medical center (km)			
<3	136709 (90.3)	127526 (88.9)	43167 (89.3)
≥3	14712 (9.7)	15854 (11.1)	5189 (10.7)

### Hospital admissions

**Figure 1:**
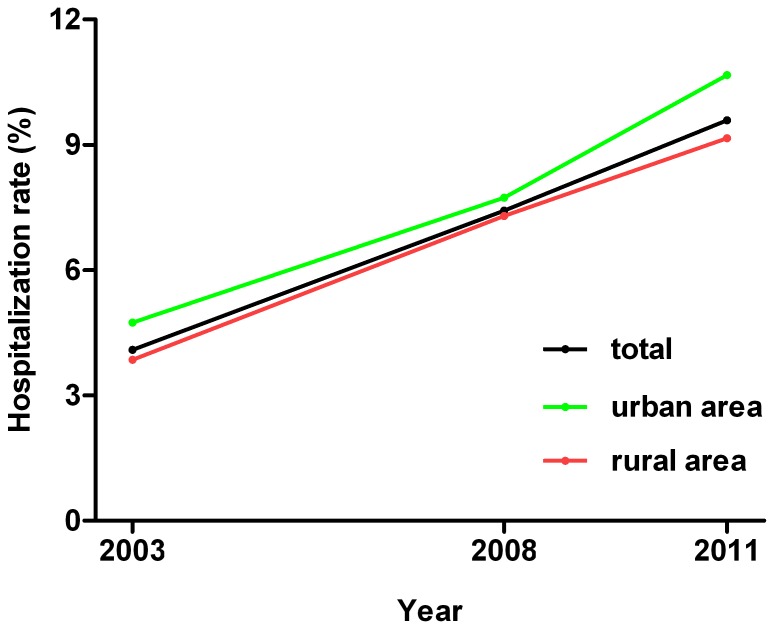
Trend of hospital admissions in total, urban and rural areas from 2003 to 2011.

Figure 1 shows the trend of hospital admissions in total, urban and rural areas from 2003 to 2011. The overall hospitalization rate was 4.1% in 2003 and rapidly increased over time, and more than doubled (9.6%) by 2011. The similar trend was observed in both urban and rural areas (*P*<0.05). The absolute difference in hospital admissions between urban and rural area decreased in 2008, then widened in 2011.

Physical illness was the main reason for hospitalization and the following was delivery. The proportion of respondents who hospitalized for illness increased over time, and decreased for delivery and injury/poisoning. Compared with rural respondents, urban respondents were more likely to be hospitalized for illness (81.0% VS 69.3% in 2011) and less likely for delivery (10.0% VS 18.3% in 2011) and injury/poisoning (3.7% VS 8.0% in 2011) (Table 2).

**Table 2:** Causes of hospitalization among urban and rural residents from 2003 to 2011, N (%).

Reasons	2003		2008		2011	
	Urban	Rural	Urban	Rural	Urban	Rural
Illness	1222 (76.9)	2176 (61.6)	1639 (77.4)	3901 (66.4)	738 (81.0)	1501 (69.3)
Delivery	225 (14.2)	800 (22.6)	292 (13.8)	1210 (20.6)	91 (10.0)	397 (18.3)
Injury/poisoning	89 (5.6)	401 (11.3)	100 (4.7)	536 (9.1)	34 (3.7)	174 (8.0)
Recovery	3 (0.2)	7 (0.2)	11 (0.5)	22 (0.4)	7 (0.8)	6 (0.3)
Birth control	3 (0.2)	24 (0.7)	4 (0.2)	18 (0.3)	3 (0.3)	5 (0.2)
Others	48 (3.0)	126 (3.6)	72 (3.4)	185 (3.2)	38 (4.2)	82 (3.8)

Table 3 shows the changes of urban-rural disparity in hospital admissions. In the aggregate, the hospitalization rate in urban residents was significantly higher than in rural residents and the *RR* (95% *CI*) of hospitalization rate was 1.23 (1.17–1.30), 1.06 (1.02–1.10) and 1.16 (1.10–1.23) in 2003, 2008 and 2011, respectively. The urban-rural disparity is considered ‘widening’ if the *RR* is moving away from 1 and ‘narrowing’ if *RR* becomes closer to 1 regardless of whether initial value is greater or less than 1. Compared to 2003, the *RR* in 2008 was moving closer to 1, the disparity was significantly narrowed by 13.8%. On the contrary, the disparity had significantly widened from 2008 to 2011.

**Table 3:** Changes of the urban-rural disparity in hospitalization rate from 2003 to 2011.

Indicator	Year	Urban (%)	Rural (%)	*RR* (95% *CI*)	Changes (%)
Total					
	2003	1966 (4.7)	4232 (3.8)	1.23 (1.17–1.30)	
	2008	3076 (7.7)	7559 (7.3)	1.06 (1.02–1.10)	−13.8
	2011	1453 (10.7)	3183 (9.2)	1.16 (1.10–1.23)	9.4
With health insurance					
	2003	1380 (5.8)	762 (4.2)	1.39 (1.27–1.51)	
	2008	2644 (8.7)	7250 (7.5)	1.17 (1.12–1.22)	−15.8
	2011	1418 (11.3)	3137 (9.2)	1.22 (1.15–1.30)	4.3
Without health insurance					
	2003	586 (3.3)	3470 (3.8)	0.88 (0.81–0.96)	
	2008	432 (4.6)	309 (4.8)	0.94 (0.82–1.09)	6.8
	2011	35 (3.4)	46 (6.5)	0.52 (0.34–0.79)	−44.7

The analyses were repeated for those with and without health insurance separately (Table 3). The urban-rural disparity depended on the health insurance status. For those with insurance, urban residents had a significantly higher hospitalization rate than their rural counterparts (5.8% VS 4.2% in 2003, 8.7% VS 7.5% in 2008 and 11.3% VS 9.2% in 2011). The urban-rural disparity significantly became narrower in 2008 compared to that in 2003 and the *RR* in 2008 decreased by 15.8%. For those without health insurance, the hospitalization rate was lower in urban than in rural area, with *RR*: 0.88 (95% CI: 0.81–0.96) in 2003 and 0.52 (95% CI: 0.34–0.79) in 2011. The urban-rural disparity became more extreme in 2011 as the *RR* moved further away from 1, implying that the urban-rural disparity significantly widened from 2008 to 2011. The percentage change in *RR* was −44.7%. Those without health insurance had a substantially lower hospitalization rate than those with health insurance.

### Medical expenditure

Table 4 shows the values of six medical expenditure measures. Among those measures, hospitalization direct cost, indirect cost, reimbursement amount and reimbursement rate increased while the proportions of hospitalization direct and indirect cost to the total consumer spending decreased over time (*P*<0.0001). The increases from 2003 to 2011 in the reimbursement amount (8.9 folds) and reimbursement rate (10.0 folds) in rural area were higher than those in urban area (1.8 folds for reimbursement amount and 1.6 folds for reimbursement rate). All the measures except the proportion of hospitalization indirect cost to the total consumer spending showed higher value in urban respondents than rural respondents. The 95% *CI*s of the difference between urban and rural areas are also shown in Table 4. The urban-rural disparities in hospitalization direct cost (2327.7 RMB in 2003 and 3273.1 RMB in 2008) and reimbursement amount (1846.7 RMB in 2003 and 2223.1 RMB in 2008) significantly widened, and significantly narrowed to 2598.2 RMB and 2047.6 RMB in 2011, respectively. The disparity in reimbursement rate significantly narrowed from 2003 (30.4%) to 2008 (11.2%). The same pattern was observed in the proportion of hospitalization direct cost to the total consumer spending from 2008 (6.8%) to 2011 (2.4%).

**Table 4:** Medical expenditure of urban and rural respondents who reported hospitalization from 2003 to 2011 (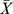).

Medical expenditure	Year	Total	Urban	Rural	Urban-rural disparity		
					Difference	Lower limit	Upper limit
HDC (RMB)	2003	3114.5	4754.6	2426.9	2327.7	2051.3	2604.1
	2008	3532.6	5961.6	2688.5	3273.1	3016.3	3530.0
	2011	4712.8	6556.1	3957.9	2598.2	2094.3	3102.1
HIC (RMB)	2003	360.5	469.0	315.0	154.0	114.9	192.9
	2008	443.7	519.6	417.3	102.3	62.1	142.5
	2011	600.8	710.1	556.0	154.1	53.9	254.3
Reimbursement amount (RMB)	2003	727.0	2028.2	181.5	1846.7	1722.0	1971.4
	2008	1343.7	2993.5	770.4	2223.1	2090.5	2355.8
	2011	2207.1	3659.7	1612.1	2047.6	1767.3	2327.9
Reimbursement rate (%)	2003	13.5	34.9	4.5	30.4	29.0	31.8
	2008	37.3	45.6	34.4	11.2	9.9	12.5
	2011	48.3	56.2	45.1	11.1	9.3	12.9
The proportion of HDC to the total CS (%)	2003	25.7	29.6	24.1	5.5	4.3	6.7
	2008	22.3	27.4	20.5	6.9	5.9	7.8
	2011	22.3	24.1	21.6	2.5	1.0	3.9
The proportion of HIC to the total CS (%)	2003	3.4	2.9	3.5	−0.6	−0.9	−0.3
	2008	3.2	2.5	3.4	−0.9	−1.2	−0.7
	2011	2.9	2.4	3.1	−0.7	−1.0	−0.4

Abbreviation: HDC, hospitalization direct cost; HIC, hospitalization indirect cost; CS, consumer spending; RMB, Renminbi.

## Discussion

The data from the China Third NHSS, the Fourth NHSS and the national health-care reform phased assessment survey show that health-care system reform had made great achievements. The health insurance coverage had been continually expanding from 27.7% in 2003 to 96.4% in 2011 among respondents aged 15 years or older. After accounting for data clustering, hospital admissions substantially improved in both urban and rural areas, and the urban-rural disparity significantly narrowed. Of the six medical expenditure measures, a rapid increase in the reimbursement amount and reimbursement rate was observed in rural residents. As a result, the urban-rural disparities in reimbursement rate and the proportion of hospitalization direct cost to the total consumer spending became significantly narrower.

The huge increases in life expectancy at birth were seen over the last decades. In 2010, Chinese life expectancy reached 74.83 years, [6] and accompanied with an aging population. In addition, many risk factors related to chronic diseases, such as the change of dietary pattern and life style have led to a sharp rise of chronic diseases in the population. [6], [27] The prevalence rate of chronic diseases among urban residents increased from 17.7% to 20.5% and from 10.5% to 14.0% among rural residents between 2003 and 2008. [28] The proportion of urban and rural respondents who hospitalized for illness increased over time, indicating that growing health service needs improved the health service utilization.

The improvement of hospital admissions was also related to unceasing improvement of the medical security system and reimbursement policies mainly focusing on inpatient coverage. Major narrowing of urban-rural disparities in hospital admissions and reimbursement rate occurred between 2003 and 2008, before the initiation of the deepening health-care system reform in 2009. Since 2003, the government has developed policies with the health budget that favors rural areas. The financial support and medical security system in rural areas have been improved step by step due to the steady development of NRCMS. The funding level of NRCMS increased from 50 RMB in 2004 to 246.2 RMB in 2011, and the proportion of participation increased from 75.2% to 97.5%. [6], [10] The reimbursement rate among rural respondents who were hospitalized in 2011 was 10.0 times that of 2003, much greater than that among urban respondents (1.6 times). The improvement of the medical security system has enhanced the medical security level and largely eased the economic burden of rural residents.

In 2009, further reform was proposed by the central government, aiming to improve the basic medical security system. [29] The country launched the URBMI and all university students were added under the coverage of URBMI. By 2010, government subsidies for NRCMS and URBMI increased to 120 RMB per person. The hospital admissions and reimbursement rate of urban and rural respondents increased along with the reform of the new medical security system. However, the trend of urban-rural disparity in hospital admissions slightly widened between 2008 and 2011. During the period, the growth amplitude of reimbursement rate among urban and rural respondents was the same.

The increase in payment ability of residents and fund into health-care system reform was another key factor which improved health service utilization. Since reform and opening up, the annual income both in urban and rural families has grown rapidly. The average annual per capita income of urban and rural residents was 21809.8 RMB and 6977.3 RMB in 2011 which was 2.6 times and 2.7 times that of 2003, respectively. [6] The data from recent 20 years show that Chinese total health expenditures have kept pace with the growth of the national economy, or slightly faster. The total health expenditure in China including government, social and individual health expenditure was 2.43 trillion RMB which was 3.7 times that of 2003. Of the total health expenditure, the proportion of individual health expenditure decreased from 40.4% in 2008 to 34.9% in 2011, in comparison with 55.9% in 2003, a drop of 21% during the 8 year period. [6] The decline of the proportion of hospitalization direct cost to the total consumer spending was faster in urban than in rural residents, indicating that urban-rural disparity in this proportion had significantly narrowed from 2003 to 2011. The enhanced capability of basic medical and health service have significantly promoted the development of equity and accessibility of medical and health service in China.

Although some progress has been made due to the recent Chinese health-care system reform, still some problems need to be tackled. The reimbursement rate for inpatients rapidly increased from 2003 to 2011, but the rate was under 50%, suggesting that the burden of disease was still heavy for residents. Because the pay cost standard of NRCMS was low and it only covered major illnesses, compared with UEBMI and URBMI, the reimbursement cap for NRCMS was lower and the coverage was narrower. The reimbursement rate of rural inpatients was 10% lower than that of urban inpatients. These findings are consistent with the results of previous studies. [23], [30] This study also found that urban respondents without health insurance were less likely to be hospitalized than their rural counterparts. Such urban-rural disparity significantly widened over the study period. It reminds us that the urban families without health insurance are more vulnerable. To solve these problems, the government should redesign reasonable multi-channel funding mechanisms to increase the funding level and reimbursement rate. The central and local governments should further increase funds to overcome inequality in the current levels of insurance due to regional differences in local economy. The welfare institutions and enterprises are encouraged to participate in medical care financing. Individual financing is suggested varying with the amount of income rather than the same funding level and more payment will gain more reimbursement. There is a great need to coordinate different health insurance systems and develop a uniform standard health insurance system for urban and rural residents. For example, URBMI and NRCMS can first use uniform standard to implement jointly and then gradually combine with the other medical insurances. In addition to the universal coverage of health insurance, the medical assistance system should be improved to satisfy the need of basic medical and health service for disadvantaged groups. Increasing the assistance funds, decreasing or canceling the deductibles and canceling the limitation on diseases which can be reimbursed will largely improve the medical security of disadvantaged groups. Furthermore, the residents who have special financial difficulties should participate in the health insurance through government funding.

Several issues should be considered in the interpretation of the findings. First, the sample frame of the Third NHSS was compared with the census in 2000 and suggested that the sampling method was adequate to generate a nationally representative sample. Therefore, the sample frame was still used in the Fourth NHSS and the survey in 2011, whereas several areas were adjusted because of the changes in those administration areas. Although the sample frame in three surveys was similar, 60 households in 2008 and 20 households in 2011 in each village or neighborhood committee were randomly selected again. The questionnaires in the Third and Fourth NHSS did not have precisely the same structure, and the questionnaire in the 2011 survey which aimed to monitor key indicators after the national health-care reform was the subset of the questionnaire in the Fourth NHSS. Second, hospital admissions and medical expenditure in this study were collected through the recall of respondents, so the results may be influenced by possible recall bias. Third, due to the limitation of the sample, the trend of urban-rural disparities was not assessed at the same time interval. However, the China fifth NHSS in 2013 had been completed and we will continue to focus on the trend of urban-rural disparities in health service utilization.

## Conclusions

In the context of recent health-care system reform in China, the health insurance coverage has been continually expanding and health service utilization has been substantially improved. The development of the New Rural Cooperative Medical Scheme has effectively narrowed the urban-rural disparities in hospital admissions and reimbursement rate. Although some progress has been made in Chinese health-care system reform, the reimbursement rate is still low and the current levels of insurance vary with different medical security systems and local economy. Thereby, the government should further increase the funds into health care, increase reimbursement rate, develop a uniform standard health insurance system for urban and rural residents and improve the medical assistance system.
